# Exploration of burnout and imposter phenomenon among dermatology resident doctors in the UK

**DOI:** 10.1093/skinhd/vzag098

**Published:** 2026-07-31

**Authors:** Honey Brandy Frimpong-Manso, Bisam-Ul Haq, Aparna Potluru, Rhianne Foley, Sidra Khan, Jonathan Guckian

**Affiliations:** General Internal Medicine, Mid and South Essex ICS, Basildon, UK; Department of Dermatology, Churchill Hospital, Oxford University Hospitals, Oxford, UK; Department of Dermatology, Queen Elizabeth Univeristy Hospital, NHS Greater Glasgow and Clyde, Glasgow, UK; School of Medicine, University of Sunderland, Sunderland, UK; Withington Community Hospital, Manchester, UK; Department of Dermatology, Leeds Teaching Hospitals NHS Trust, Leeds, UK

## Abstract

**Background:**

Dermatology training is highly competitive, yet UK-specific data on trainee wellbeing are limited. Burnout describes emotional exhaustion and reduced professional fulfilment due to chronic stress, while imposter phenomenon (IP) reflects persistent self-doubt despite objective competence.

**Objectives:**

To examine the prevalence and drivers of burnout and IP among UK dermatology registrars.

**Methods:**

A national questionnaire survey was distributed to UK dermatology trainees in May 2025. Burnout was assessed using the Emotional Exhaustion (EE) subscale of the Maslach Burnout Inventory, and IP using validated scales. Free-text responses explored drivers of burnout and IP and protective factors, and were analysed using inductive thematic analysis.

**Results:**

Eighty-four trainees responded [74% women (*n* = 62/84)]. Significant burnout (EE >13) was identified in 44% (*n* = 37/84). Trainees with ­burnout reported significantly higher IP scores than those without (mean 26 vs. 22; *P* = 0.005). Burnout was strongly associated with poor work–life integration (*P* < 0.001) and thoughts of leaving the specialty [49% (*n* = 41/84) vs. 17% (*n* = 14/84); *P* = 0.001]. There were no significant differences between burnout groups by age, sex or ethnicity. Qualitative analysis identified five themes: unattainable targets (administrative workload and Specialty Certificate Examination pressure), disrupted transitions, feelings of abandonment due to limited supervision, competing priorities and professional identity violation.

**Conclusions:**

These novel UK data demonstrate that burnout and IP are common and closely related among dermatology registrars. Prevalence was comparable to that reported in international literature. Drivers appear to be predominantly structural rather than demographic, particularly administrative burden, high-stakes assessment and inconsistent access to support. System-level interventions, including protected self-development time and increased examination sittings, may help support the workforce. These findings provide an important baseline for future policy development and research into trainee wellbeing in UK dermatology.

## Teaser

Burnout among medical trainees is increasingly recognized as an important determinant of professional wellbeing and workforce stability. While dermatology is frequently regarded as a specialty characterized by favourable working hours and less acute clinical presentations, this perception may obscure important stressors experienced during training. This study, based on the largest UK national dermatology resident doctor survey, presents findings that suggest burnout and dissatisfaction with work–life balance remain relevant concerns among trainees. Furthermore, the highly competitive nature of this specialty fuels the imposter phenomenon experienced by some trainees. Pressures related to clinical service demands, administrative burden and academic expectations as well as career progression contribute to emotional exhaustion, thus we present a closer examination of the structural and cultural factors that influence dermatology trainee wellbeing within the national training programme.

What is already known about this topic?

- Burnout is common among postgraduate medical trainees and is associated with emotional exhaustion, reduced wellbeing and workforce attrition.
- Dermatology trainees internationally report high levels of burnout and imposter phenomenon, particularly in competitive training environments.
- Administrative burden and long working hours are recognized contributors, although UK-specific data in dermatology are limited.

What does this study add?

- This is the largest UK study to date to examine burnout and imposter phenomenon among dermatology registrars.
- A strong association exists between burnout and imposter phenomenon, independent of demographic factors.
- The study identifies UK-specific training pressures, including administrative workload, portfolio demands, limited supervision and the Specialty Certificate Examination, as key drivers likely to impact wellbeing and professional identity.

The transition into specialty training marks a pivotal juncture for UK medical trainees entering dermatology, a process known to be associated with intense competition and academic rigour.^1^ Trainees navigate a host of stressors, including high volumes of administrative workload, breadth of clinical exposure and the requirement to complete the Specialty Certificate Examination (SCE), all while balancing personal responsibilities. It has been established in other medical and surgical specialties that such pressures contribute to mounting psychological distress and burnout.^2–4^ These challenges may erode work–life balance and reduce job satisfaction, and can negatively affect academic outcomes and long-term professional growth.^5^

Burnout is a psychological syndrome characterized by emotional exhaustion, depersonalization and a diminished sense of personal accomplishment, often caused by prolonged exposure to stressors.^6^ Reported rates of burnout during postgraduate medical training vary widely, ranging from 18% to 84%, reflecting the intense pressures faced by resident doctors.^7,8^ It has been consistently linked to adverse outcomes in both professional performance and overall health.^6^ In a 2018 study of Canadian dermatology residents, 52% experienced depressed mood, 20% had thoughts of self-harm, 27% had anxiety and 45% reported problematic alcohol use.^9^

Meanwhile, imposter phenomenon (IP), also known as imposter syndrome, refers to persistent self-doubt and the fear of being exposed as a fraud, despite evident achievements.^10^ IP can undermine a physician’s professional identity development and adversely affect their personal wellbeing.^11^ IP remains underexplored in medicine, although emerging research in medical students and primary care trainees suggests a connection to burnout and psychiatric comorbidities.^12,13^

In 2025, the General Medical Council (GMC) identified that over a fifth of trainees were at high risk of burnout, and over half described their work as emotionally exhausting to a very high or high degree.^14^ Despite these well-documented challenges there is a lack of research exploring the lived experiences of dermatology registrars in the UK, leaving a critical gap in our understanding of how burnout and IP manifests in dermatology. Existing data among dermatology residents are largely derived from Canadian and US-based studies, where burnout rates have reached as high as 69%.^5,10^ However, these findings may not translate directly to the UK context, given differences in training structures, healthcare systems and cultural expectations.

There is therefore a pressing need to deepen our understanding of the experiences of dermatology trainees in the UK. This study aimed to illuminate the interplay between burnout, IP and trainee wellbeing. Identifying key contributing factors, as well as protective influences, may inform the development of targeted interventions to support dermatology registrar performance, mental health and long-term retention.

## Materials and methods

The phenomena explored in this study were the prevalence, causes and consequences of burnout and IP among dermatology trainees. Inclusion criteria featured resident doctors who had had a National Training Number in UK dermatology training at the point of data collection in May 2025. IP and burnout have been identified among Specialty, Associate Specialist and Specialist doctors; however, given the variability of educational context when compared with trainees, they were excluded from this study.

Combining key elements of the Maslach Burnout Inventory and the validated Quality of Life Five Dimensions Index,^5^ validated tools in assessing burnout, we created a 35-item survey to address our research question. Demographic information such as age, sex, relationship status and stage of training were also collected. To enhance brevity and response rates, the survey focused on emotional exhaustion rather than the complete burnout inventory. The final questions in the survey were chosen based on piloting and consensus among the authors. Open-ended free-text questions explored protective and perpetuating factors influencing burnout, IP and future career decision-making.

Following ethical approval from the University of Sunderland, participants were recruited via British Association of Dermatologists trainee mailing lists, social media and word of mouth.

Statistical analysis was performed using R software (version 4.3; R Foundation for Statistical Computing, Vienna, Austria). Statistical significance was set at a two-tailed *P*-value of < 0.05. High burnout was defined by a median split of Emotional Exhaustion (EE) scores, with values above the median classified as high burnout. Bivariate analyses compared characteristics between high-burnout and nonburnout groups using χ^2^ or Fisher’s exact tests for categorical variables and independent *t*-tests or Wilcoxon rank-sum tests for continuous variables.

A qualitative analysis of free-text responses was conducted using Braun and Clark’s inductive thematic analysis approach.^15^ This provided a systematic and rigorous framework for identifying patterns and meanings within the dataset. The six-phase process began with researchers familiarizing themselves with participants’ responses, generating codes and the development of recurring themes, which were subsequently reviewed and refined by senior researchers. All themes were refined to capture the essence of participants’ sentiments and illustrated with key quotes that best exemplify them.

## Results

The study cohort comprised 84 dermatology trainees. Of the respondents, 74% (*n* = 62/84) were female, 50% (*n* = 42/84) identified as White and the majority were 30–35 years old. Additionally, 39% (*n* = 33/84) were married and 33% had children or dependents. Trainees with burnout had statistically significant higher IP scores than those without (mean 26 vs. 22; *P* = 0.005), indicating a strong association between burnout and IP in this group.

In this population, 44% (*n* = 37/84) of participants met the criteria for high burnout (Figure 1). The top factors influencing burnout for respondents included managing administrative (admin) load, meeting academic needs and sitting the SCE examination (Figure 1).

**Figure 1:**
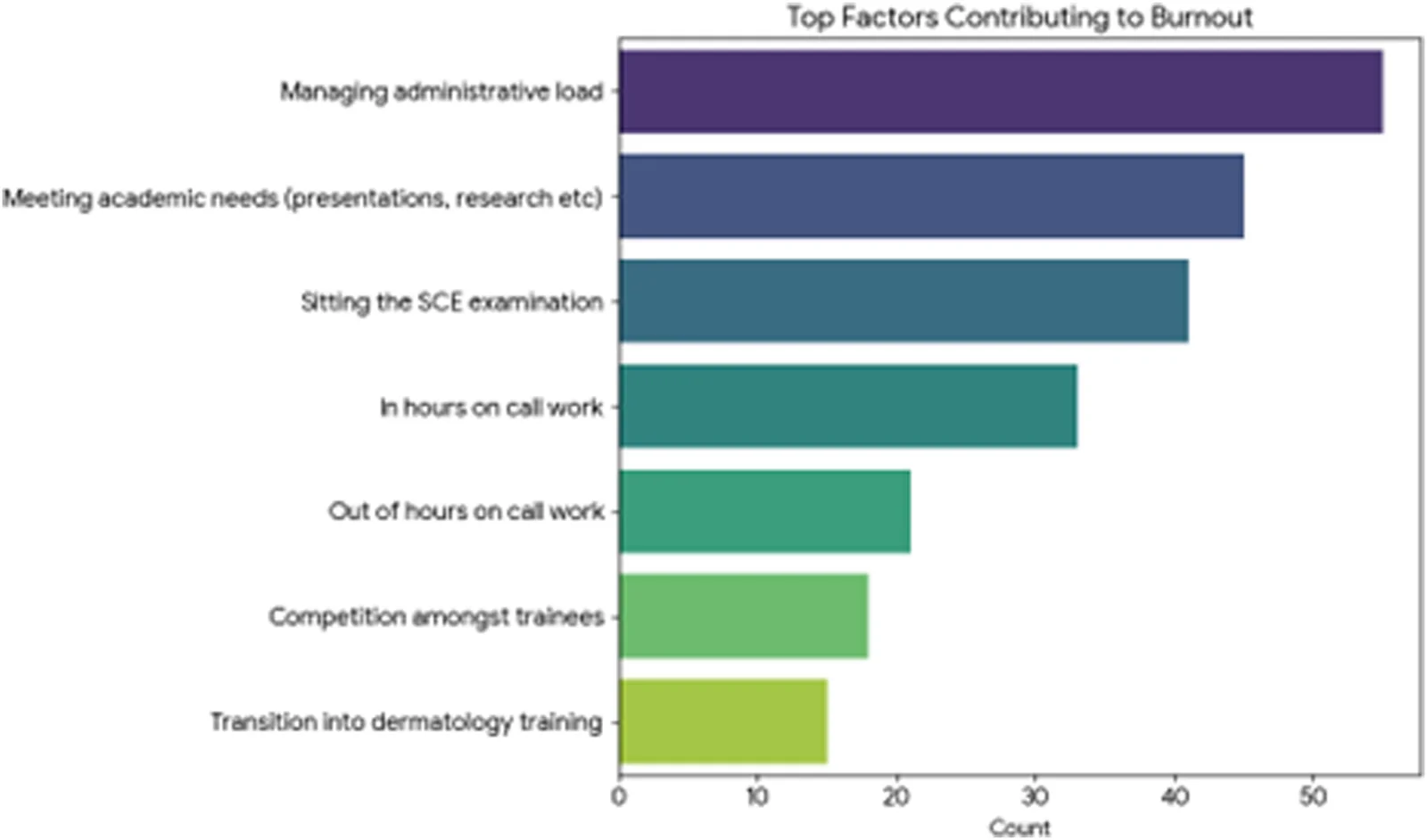
Top factors contributing to burnout. SCE, Specialty Certificate Examination.

Analysis of protective factors against burnout revealed that structural and administrative changes were most highly valued. Specifically, more scheduled admin time was the most frequently cited factor (*n* = 55), followed by increased SCE support and increased study leave/funding (Figure 2).

**Figure 2:**
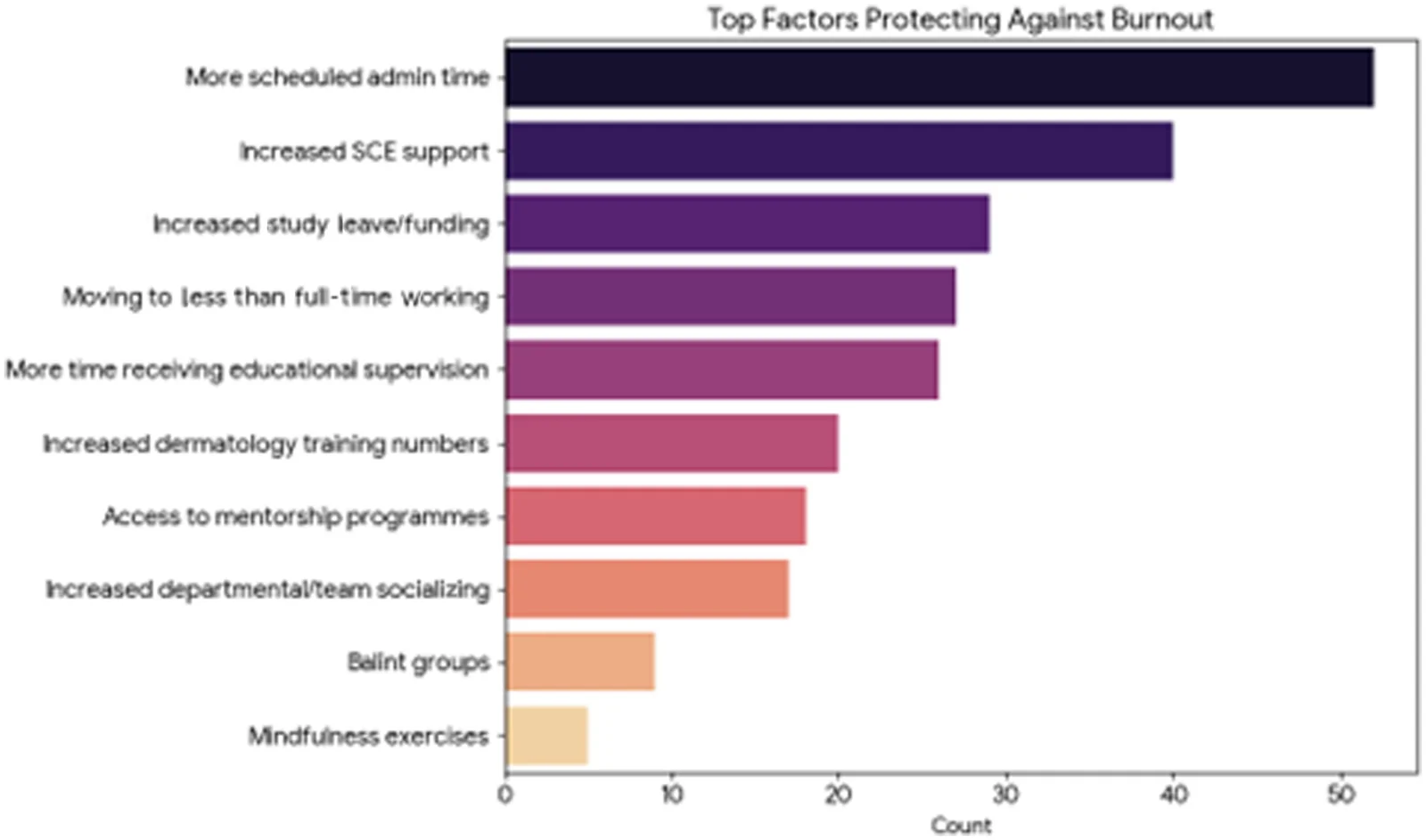
Top factors protecting against burnout.

The analysis revealed no statistically significant differences between the high burnout and nonburnout groups across any measured demographic variable, including age (*P* = 0.30), sex (*P* = 0.30), ethnicity (*P* = 0.60) and stage of training (*P* > 0.90) (Appendix S1; see Supporting Information). These findings suggest that burnout was not significantly associated with the demographic factors within our study population.

Trainees meeting our threshold for burnout (EE score >13) exhibited significantly elevated mean (SD) IP scores compared with the nonburnout cohort [26 (7) vs. 22 (6)], with Figure 3 showing a clear upward shift in the median and interquartile range for the burnout group. Despite this strong psychological correlation, the burnout index itself remained relatively consistent across demographic categories; male and female trainees reported nearly identical median scores (approximately 3.0), indicating that sex was not a significant differentiator for burnout levels within this population (Figure 4).

**Figure 3:**
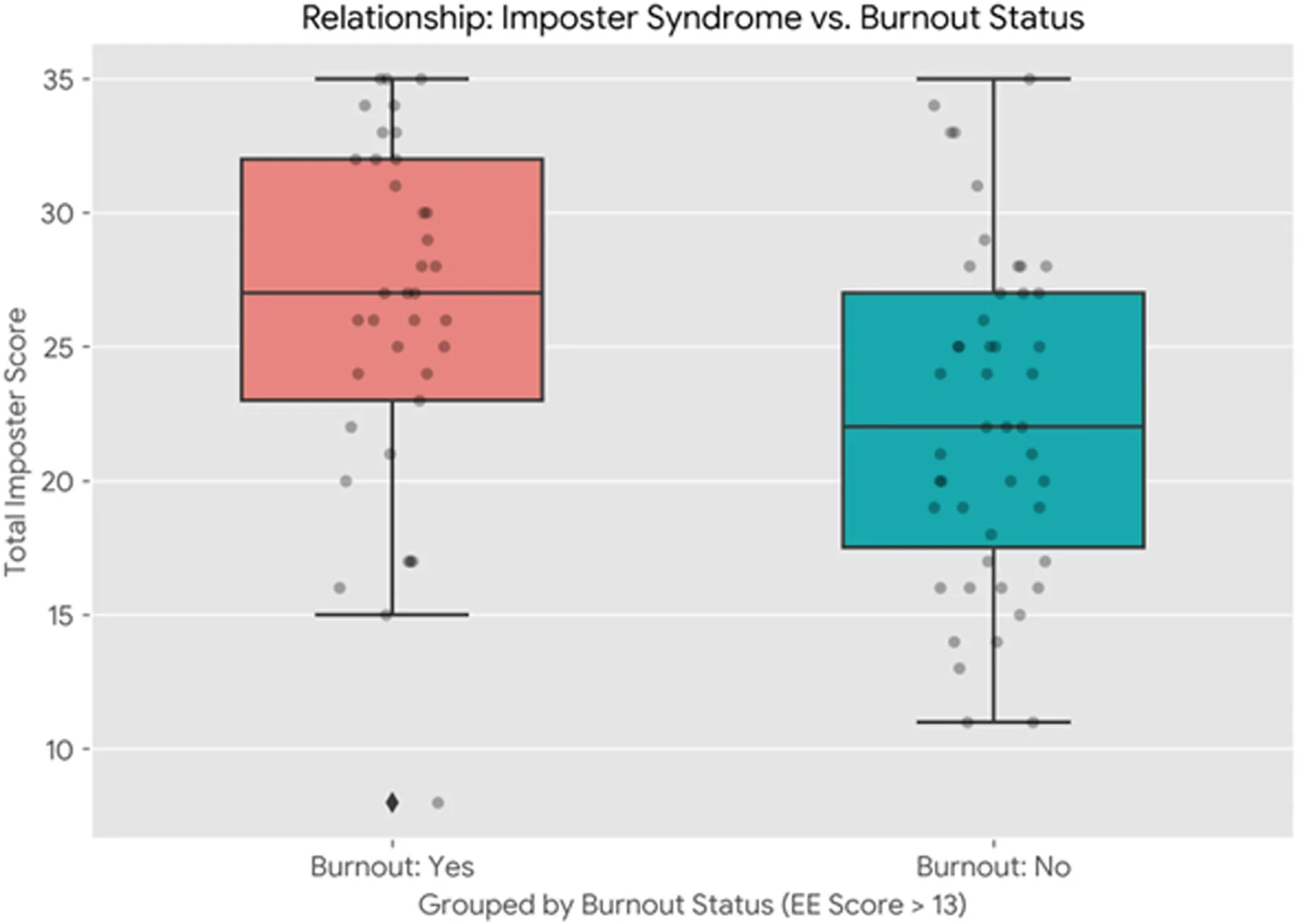
Relationship between imposter syndrome and burnout status. EE, Emotional Exhaustion.

A highly significant association was identified between high burnout and difficulties with work–life integration (see Appendix S1). Trainees with high burnout reported widespread dissatisfaction with their work–life balance; 57% (*n* = 48/84) were unsatisfied or very unsatisfied, in stark contrast to the nonburnout group, where a majority (65%; *n* = 55/84) reported being satisfied or very satisfied (*P* < 0.001). This theme extended to personal wellbeing, with the high-burnout group consistently reporting that training demands significantly interfered with personal activities. Notably, 85% (*n* = 71/84) of trainees who were burned out agreed or strongly agreed that training made self-care difficult, compared with only 15% (*n* = 13/84) of their peers who were not burned out. This pattern of difficulty was consistent across all measured activities, including seeing friends and family, exercising, getting adequate sleep and maintaining relationships (all *P* < 0.001).

**Figure 4:**
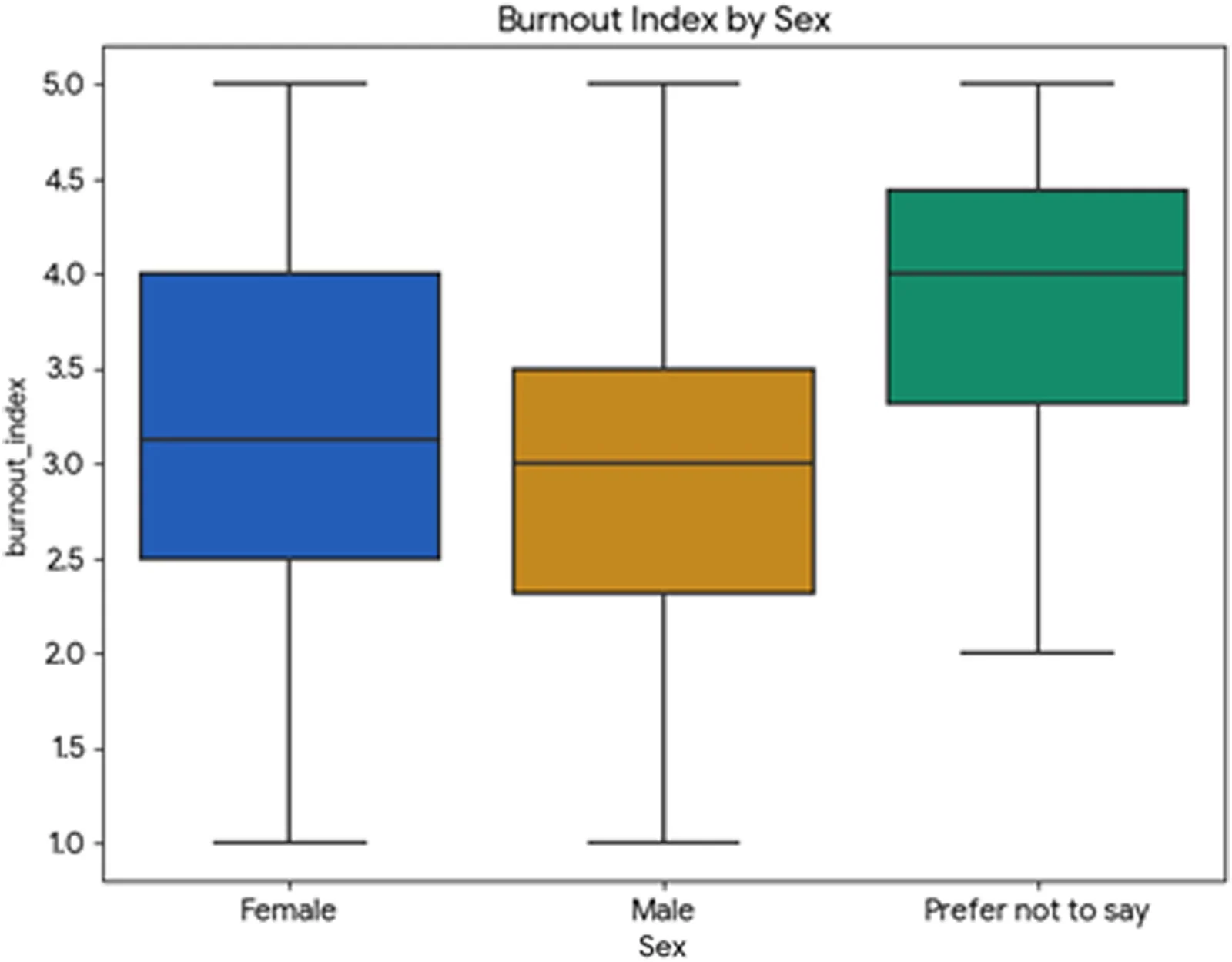
Burnout by sex.

Burnout was found to be strongly associated with career dissatisfaction and thoughts of leaving the specialty (see Appendix S1). Trainees in the high burnout group were significantly more likely to have considered leaving dermatology compared with their colleagues who were not burned out [49% (*n* = 41/84) vs. 17% (*n* = 14/84); *P* = 0.001]. This finding was consistent with career choice regret, as a significantly higher proportion of trainees who were burned out were unsure whether they would apply to dermatology again [44% (*n* = 37/84) vs. 13% (*n* = 11/84); *P* = 0.001]. Furthermore, when asked about quitting or switching careers, 40% (*n* = 34/84) of the high-burnout group strongly agreed, in contrast to 8% (*n* = 7/84) of the nonburnout group (*P* = 0.007).

### Qualitative analysis

Rich qualitative responses revealed five overarching themes: unattainable targets, disrupted transitions, feeling of abandonment, competing priorities and competing colleagues, and professional identity violation.

#### Theme 1: unattainable targets

Trainees described heavy workloads driven by large patient volumes, diverse clinical demands and constant time pressure. They were expected to complete ‘a large volume’ of academic tasks, research, teaching, audits and portfolio work, often ‘in a single admin session’. Lack of administrative time meant work ‘bleeding into personal time’, creating an ‘invisible workload’, reducing rest and recovery. Increasing admin time was commonly suggested.

Many felt overwhelmed balancing clinical duties with on-calls, which were described as ‘nonstop’ and requiring trainees to be ‘on [their] toes at all times’. Increasing training numbers was frequently proposed to share the workload, although many felt this was ‘not feasible’.

Studying for the SCE required ‘countless hours’ outside work while managing family commitments. The ‘overwhelming’ syllabus and high pass marks made success feel out of reach, leaving trainees ‘exhausted’ and ‘demoralized’, and considering leaving dermatology. Some reported significant personal sacrifices, including missing family milestones, and one described resitting the exam as something that ‘almost broke’ them. Increasing the number of annual sittings was seen as a clear way to lessen ‘the pressure’.

#### Theme 2: disrupted transitions

Inconsistent support and expectations across trusts created a ‘shocking’ ‘baptism of fire’ for new trainees. Some felt that IP persisted despite support, driven by internal ‘perfectionistic tendencies’ that were ‘exhausting’. Supportive senior staff reduced uncertainty, and ‘meaningful positive feedback’ was viewed as essential. Its absence heightened IP. ‘Open’, ‘reassuring’ discussions acknowledging IP helped create a culture of belonging.

#### Theme 3: feelings of abandonment

Many registrars felt like ‘just service provision’, with some ‘effectively supervising themselves’ due to consultant workload. Lack of feedback fuelled uncertainty and IP, and trainees felt they had to ‘train [themselves] through reading all the time… spat out at the other end’. They called for protected supervision and teaching time, especially at ST3, with senior availability to ‘discuss every case’.

Service was widely seen as prioritized over training. Increasing senior staff was considered essential both for service delivery and ‘genuine educational value’. Several suggested Balint groups as a supportive, reflective space.

#### Theme 4: competing priorities and competing colleagues

Limited free time and competing demands left trainees feeling ‘guilt’ and ‘imbalanced’. Home responsibilities were reframed as ‘wasting my time when I am not endeavouring to further my career in dermatology’, contributing to ‘doubt’ and shame. Some sought better balance through less than full-time training. A competitive culture also fuelled IP, as trainees viewed peers as ‘impressive’ and felt they were ‘constantly being compared’.

#### Theme 5: professional identity violation

Respondents frequently encountered stereotypes, including dermatology being a ‘dermaholiday’, or that dermatologists ‘just sit at home’. These misconceptions were ‘deeply demoralizing’, contributed to ‘isolation’, and undermined self-worth. Feeling undervalued was linked to attitudes from nondermatologists, who felt dermatologists ‘should be paid less’, and to training structures such as rotations, which left trainees feeling like ‘temporary staff’ who ‘don’t fit in’. Despite being ‘highly qualified doctors’, many felt ‘infantilized’, contrasting with their identities outside work, including academIc and parenting roles.

## Supplementary Material

vzag098_Supplementary_Data

## Discussion

A significant proportion of dermatology residents surveyed met the criteria for burnout. Closely tied to IP, burnout prompted many to contemplate leaving or changing careers. Contributing factors included limited senior support, high administrative burdens and unattainable performance targets, all of which compromised work–life balance. IP further eroded confidence and contributed to a sense of professional identity violation.

Recent GMC data demonstrated that 20% of postgraduate trainees were ‘at high risk of burnout’ and 50% experienced emotional exhaustion to a high or very high degree from their work.^14^ However, the survey focuses on burnout in acute specialties where nightshifts and on-call work are common, so burnout in dermatology, which is often linked to ongoing cognitive and administrative pressures, may be overlooked.^14^ Our study suggests that dermatology registrars surveyed are experiencing burnout to a high degree, comparable to national cohorts. Internationally, studies in the USA have reported the prevalence of burnout among US dermatology residents to range from 29% (*n* = 71)^16^ to 69% (*n* = 180),^5^ with a Spanish study reporting a rate of 23% of dermatology residents at moderate risk of burnout (*n* = 48).^17^

There are limited studies on IP in dermatology trainees, but it is highly researched among general medical professionals. A study in the US discussed IP in dermatologists and residents, drawing a significant correlation between the competitiveness of applications and the level of confidence to match into the specialty.^18^ They also found no significant difference in IP between male and female participants,^18^ although throughout literature it is commonly cited that IP is higher among female individuals^19^ and IP was first described by psychologists as a concept in high-achieving women.^20^ Another US-based study described the prevalence of IP among dermatology residents at 92% in women and 85% in men.^10^ Across all studies, the competitive nature of dermatology is named as a contributing factor to high levels of IP.^10,18^

A number of theoretical models could link the reported drivers for burnout. Professional identity formation (PIF) entails how professionals internalize the beliefs, practices and core values of a profession.^21^ The results from our qualitative analysis, including the external perception of dermatology, the disrupted transition between training stages and feelings of abandonment, may all be factors that affect PIF, and this identity crisis may help explain our results. The dissonance between the expectations and realities of one’s career, and the set expectations within the curriculum vs. the mentality of ‘service provision’ competing with training needs all fuel negative experiences within training that will lead to a lack of fulfilment and demoralization of the workforce, frequently described in PIF literature.^22^

Previous studies link burnout to long hours, administrative burden and limited time for recovery.^5^ Our findings extend this by showing that UK-specific training demands, particularly portfolio requirements and preparation for the SCE, intensify these pressures and contribute to burnout and IP. Trainees described sustained high-intensity work alongside extensive unpaid academic labour, leading to exhaustion, demoralization and consideration of leaving dermatology. These experiences may be best understood through the lens of moral injury, where clinicians are unable to practise in ways that align with their professional values due to systemic constraints. As Rosenbaum^23^ argues, framing burnout as an individual failure of resilience obscures the structural conditions that erode wellbeing; our data illustrate how prolonged exposure to these constraints ultimately depletes trainees’ capacity to continue giving, with implications for both workforce retention and patient care. Moreover, dermatology trainees may relocate away from family and established support networks, contributing to social isolation and reduced wellbeing.

Based on our findings and the identified key drivers of burnout, there are several implications for both practice and policy within higher speciality training. Firstly, we advocate for the introduction of self-development time, separate from admin time and comparable to that provided during foundation and core training. This principle of protected time acknowledges the ‘invisible’ workload borne by doctors, a burden that intensifies as training progresses. At present, portfolio requirements and clinic-related administrative tasks frequently encroach upon personal time. Furthermore, we recommend that activities often perceived as extracurricular, such as poster and case presentations and organization of meetings, be formally embedded within the training curriculum and supported by dedicated time for participation. The expectation of participation in these for portfolio development, while expecting this to be completed in personal time, is not acceptable. If such activities are essential for career progression, they should be explicitly recognized as core components of training, appropriately funded in study budgets, and linked to protected time and appropriate senior support for project design and completion.

Furthermore, we strongly recommend consideration of the introduction of an additional SCE examination sitting each year. At present, only a single sitting is offered annually, and our quantitative and qualitative findings indicate that this places a substantial and disproportionate burden on trainees, generating intense pressure during preparation for a single high-stakes assessment. Expanding the number of sittings, alongside providing financial support for examination preparation and enhanced access to study leave, may meaningfully alleviate the stress experienced by trainees. The substantial transition when entering dermatology from prior roles with greater clinical independence can be particularly challenging for high-achieving trainees and contribute to IP. Targeted educational support at key transition points, including entry into higher specialty training and progression to consultant level, may help navigation of identity shifts. This may manifest as peer socialization and practical education to ‘bookend’ training.

Future research informed by this study could include extending the survey to consultants and clinical trainers. As highlighted by the GMC National Training Survey, trainers are themselves at significant risk of burnout, which may, in turn, affect the quality and consistency of supervision provided.^14^ A more in-depth qualitative exploration, such as semistructured interviews, involving trainees and trainers would allow further examination of professional identity, stereotypes and transitional challenges within dermatology higher specialty training.

A key strength of this study is the relatively high response rate, representing, to our knowledge, the largest participation achieved in a UK dermatology trainee survey to date. The use of validated instruments to assess the prevalence of burnout and IP among doctors strengthens the reliability of our findings, which are further supported by rich qualitative insights derived from open-ended survey responses (Appendix S2; see Supporting Information).

We used a selection of the scoring tool for the sake of brevity of our survey and to maximize response rates. This limited our ability to provide a wider score for burnout and fully measure subthemes such as personal accomplishment which is under-reported in many studies. We received a relatively high response rate, but, objectively, this was a small sample size, limiting the results of our study. Due to the methods of distribution, responses were susceptible to self-selection bias where those who chose to respond to our survey may have been more motivated or opinionated to share their views.

Burnout and IP are prevalent among UK dermatology registrars and are strongly associated with one another. Our findings indicate that these experiences are driven primarily by structural features of training, including high administrative workload, competing academic expectations, limited supervision and the pressures of high-stakes assessment. Qualitative data highlight how these factors disrupt professional identity development and contribute to feelings of exhaustion, demoralization and career doubt. Addressing burnout in dermatology training therefore requires system-level interventions that better align service demands, educational expectations and trainee wellbeing. Without such changes, the sustainability of the dermatology workforce and the quality of patient care may be increasingly compromised.
