# Supplementary material for: Exploration of burnout and imposter phenomenon among dermatology resident doctors in the UK
Source: Skin Health Dis. 2026 Jul 31;6(5):763–9. doi: 10.1093/skinhd/vzag098 (PMC13623432; doi:10.1093/skinhd/vzag098)
Supplement: Supplementary file 1 [file vzag098_supplementary_data.docx]

**Appendixes**

**Appendix S1 - Tables and Data**

**Table 1** - Imposter Syndrome Score by Burnout Status

| **Characteristic** | **Overall**  N = 84 | **Burnout: No**<br>N = 47 | **Burnout: Yes**<br>N = 37 | **p-value**^1^ |
| --- | --- | --- | --- | --- |
| Imposter Syndrome Score |  |  |  | **0.005** |
| Mean (SD) | 24 (7) | 22 (6) | 26 (7) |  |
| ^1^Wilcoxon rank sum test | | | | |

**Table 2 -**  Demographic and Professional Characteristics of UK Dermatology Registrars Categorised by Burnout Status.

| **Characteristic** | **Overall**  N = 84^1^ | **No**  N = 47^1^ | **Yes**  N = 37^1^ | **p-value**^2^ |
| --- | --- | --- | --- | --- |
| Age |  |  |  | 0.3 |
| < 30 | 4 (4.8%) | 4 (8.5%) | 0 (0%) |  |
| > 50 | 1 (1.2%) | 1 (2.1%) | 0 (0%) |  |
| 30-35 | 62 (74%) | 33 (70%) | 29 (78%) |  |
| 36-40 | 11 (13%) | 5 (11%) | 6 (16%) |  |
| 41-50 | 6 (7.1%) | 4 (8.5%) | 2 (5.4%) |  |
| Sex |  |  |  | 0.3 |
| Female | 62 (74%) | 34 (72%) | 28 (76%) |  |
| Male | 18 (21%) | 12 (26%) | 6 (16%) |  |
| Prefer not to say | 4 (4.8%) | 1 (2.1%) | 3 (8.1%) |  |
| Race/Ethnicity |  |  |  | 0.6 |
| Arab | 1 (1.2%) | 1 (2.1%) | 0 (0%) |  |
| Asian | 25 (30%) | 14 (30%) | 11 (30%) |  |
| Black African | 4 (4.8%) | 3 (6.4%) | 1 (2.7%) |  |
| Caucasian / White | 42 (50%) | 25 (53%) | 17 (46%) |  |
| Chinese | 1 (1.2%) | 1 (2.1%) | 0 (0%) |  |
| Don’t want to be identifiable | 1 (1.2%) | 0 (0%) | 1 (2.7%) |  |
| Hispanic | 1 (1.2%) | 1 (2.1%) | 0 (0%) |  |
| Middle Eastern | 1 (1.2%) | 0 (0%) | 1 (2.7%) |  |
| Mixed | 4 (4.8%) | 1 (2.1%) | 3 (8.1%) |  |
| Mixed middle eastern/caucasian | 1 (1.2%) | 0 (0%) | 1 (2.7%) |  |
| Prefer not to say | 2 (2.4%) | 1 (2.1%) | 1 (2.7%) |  |
| White Irish | 1 (1.2%) | 0 (0%) | 1 (2.7%) |  |
| Stage of Training |  |  |  | >0.9 |
| Out of training | 5 (6.0%) | 3 (6.4%) | 2 (5.4%) |  |
| ST3 | 20 (24%) | 13 (28%) | 7 (19%) |  |
| ST4 | 22 (26%) | 11 (23%) | 11 (30%) |  |
| ST5 | 17 (20%) | 9 (19%) | 8 (22%) |  |
| ST6 | 20 (24%) | 11 (23%) | 9 (24%) |  |
| Relationship Status |  |  |  | 0.2 |
| Married | 33 (39%) | 16 (34%) | 17 (46%) |  |
| Partnered | 27 (32%) | 18 (38%) | 9 (24%) |  |
| Separated | 2 (2.4%) | 0 (0%) | 2 (5.4%) |  |
| Single | 22 (26%) | 13 (28%) | 9 (24%) |  |
| Has Children/Dependents | 28 (33%) | 13 (28%) | 15 (41%) | 0.2 |
| ^1^n (%) | | | | |
| ^2^Fisher's exact test; Pearson's Chi-squared test  Data are presented as count (n) and **column percentage (%)**, representing the proportion of participants within each outcome group. | | | | |

**Table 3** - Association Between Burnout Status and Work-Life Integration Among UK Dermatology Registrars.

| **Characteristic** | **Overall**  N = 84^1^ | **Non burnout?No**  N = 47^1^ | **Burnout?Yes**  N = 37^1^ | **p-value**^2^ |
| --- | --- | --- | --- | --- |
| Satisfaction with Work-Life Balance |  |  |  | <0.001 |
| Very Unsatisfied | 6 (7.1%) | 0 (0%) | 6 (16%) |  |
| Unsatisfied | 20 (24%) | 5 (11%) | 15 (41%) |  |
| Neutral | 22 (26%) | 11 (23%) | 11 (30%) |  |
| Satisfied | 30 (36%) | 25 (53%) | 5 (14%) |  |
| Very Satisfied | 6 (7.1%) | 6 (13%) | 0 (0%) |  |
| Training makes it hard to see friends/family |  |  |  | <0.001 |
| Strongly Disagree | 13 (15%) | 12 (26%) | 1 (2.7%) |  |
| Disagree | 17 (20%) | 13 (28%) | 4 (11%) |  |
| Neutral | 22 (26%) | 13 (28%) | 9 (24%) |  |
| Agree | 20 (24%) | 8 (17%) | 12 (32%) |  |
| Strongly Agree | 12 (14%) | 1 (2.1%) | 11 (30%) |  |
| Training makes it hard to exercise |  |  |  | <0.001 |
| Strongly Disagree | 12 (14%) | 12 (26%) | 0 (0%) |  |
| Disagree | 11 (13%) | 9 (19%) | 2 (5.4%) |  |
| Neutral | 20 (24%) | 15 (32%) | 5 (14%) |  |
| Agree | 28 (33%) | 9 (19%) | 19 (51%) |  |
| Strongly Agree | 13 (15%) | 2 (4.3%) | 11 (30%) |  |
| Training makes it hard to get adequate sleep |  |  |  | <0.001 |
| Strongly Disagree | 13 (15%) | 13 (28%) | 0 (0%) |  |
| Disagree | 23 (27%) | 19 (40%) | 4 (11%) |  |
| Neutral | 19 (23%) | 12 (26%) | 7 (19%) |  |
| Agree | 20 (24%) | 3 (6.4%) | 17 (46%) |  |
| Strongly Agree | 9 (11%) | 0 (0%) | 9 (24%) |  |
| Training makes it hard to do self-care |  |  |  | <0.001 |
| Strongly Disagree | 10 (12%) | 10 (21%) | 0 (0%) |  |
| Disagree | 20 (24%) | 16 (34%) | 4 (11%) |  |
| Neutral | 16 (19%) | 14 (30%) | 2 (5.4%) |  |
| Agree | 28 (33%) | 6 (13%) | 22 (59%) |  |
| Strongly Agree | 10 (12%) | 1 (2.1%) | 9 (24%) |  |
| Training makes it hard to maintain a relationship |  |  |  | <0.001 |
| Strongly Disagree | 21 (25%) | 18 (38%) | 3 (8.1%) |  |
| Disagree | 28 (33%) | 18 (38%) | 10 (27%) |  |
| Neutral | 17 (20%) | 7 (15%) | 10 (27%) |  |
| Agree | 12 (14%) | 4 (8.5%) | 8 (22%) |  |
| Strongly Agree | 6 (7.1%) | 0 (0%) | 6 (16%) |  |
| ^1^n (%) | | | | |
| ^2^Fisher's exact test; Pearson's Chi-squared test **Table 4 - Career Intentions and Specialty Choice Regret by Burnout Status** **Characteristic** \| **Overall** N = 84^1^ \| **No** N = 47^1^ \| **Yes** N = 37^1^ \| **p-value**^2^; Considered quitting/switching career \| \| \| \| 0.007; 1 \| 35 (42%) \| 25 (53%) \| 10 (27%) \|; 2 \| 15 (18%) \| 8 (17%) \| 7 (19%) \|; 3 \| 7 (8.3%) \| 5 (11%) \| 2 (5.4%) \|; 4 \| 8 (9.5%) \| 5 (11%) \| 3 (8.1%) \|; 5 \| 19 (23%) \| 4 (8.5%) \| 15 (41%) \|; Would apply to dermatology again \| \| \| \| 0.001; Not sure \| 22 (27%) \| 6 (13%) \| 16 (44%) \|; Yes \| 61 (73%) \| 41 (87%) \| 20 (56%) \|; Unknown \| 1 \| 0 \| 1 \|; Considered leaving dermatology \| \| \| \| 0.001; No \| 57 (68%) \| 39 (83%) \| 18 (49%) \|; Prefer not to answer \| 1 (1.2%) \| 0 (0%) \| 1 (2.7%) \|; Yes \| 26 (31%) \| 8 (17%) \| 18 (49%) \|; ^1^n (%) \| \| \| \|; ^2^Fisher's exact test; Pearson's Chi-squared test \| \| \| \| | | | | |

**Appendix S2 : Study Questionnaire Instrument**

Section 1: Participant Consent

1. I confirm that I have read the information sheet for this study. I have had the opportunity to consider the information, ask questions and have had these answered satisfactorily.

☐ Yes

☐ No

2. I understand that my participation is voluntary and that I am free to withdraw within 48 hours of my answers being collected, without giving any reason, and that my responses will not impact my dermatology training in any way.

☐ Yes

☐ No

3. I understand that the information collected about me will be used to support other research in the future, and may be shared anonymously with other researchers.

☐ Yes

☐ No

4. I agree to take part in this study.

☐ Yes

☐ No

Section 2: Demographics

5. Sex

☐ Male

☐ Female

☐ Prefer not to say

6. Age

☐ <30

☐ 30-35

☐ 36-40

☐ 41-50

☐ >50

7. Race/ethnicity

☐ Asian

☐ Black African

☐ Black Caribbean

☐ Caucasian/White

☐ Hispanic

☐ Other

8. What stage of training are you currently?

☐ ST3

☐ ST4

☐ ST5

☐ ST6

☐ Out of training

9. Which area are you currently training in?

☐ England / ☐ Scotland / ☐ Northern Ireland / ☐ Wales / ☐ Prefer not to say

+4

10. What is your relationship status?

☐ Single / ☐ Married / ☐ Partnered / ☐ Separated / ☐ Divorced

+4

11. Do you have children or other dependents?

☐ Yes / ☐ No

+1

Section 3: Wellbeing and Burnout

Responses scored on a 5-point Likert scale (1 = Strongly Disagree to 5 = Strongly Agree unless otherwise stated).

12. Currently, how satisfied are you with the balance between your personal and professional life? (1 = Extremely unsatisfied to 5 = Extremely satisfied)

+2

13. "Dermatology training is a necessary and temporary time of personal sacrifice, with great rewards in the end"

14. "Dermatology training has made it difficult for me to see my family / friends"

15. "Dermatology training has made it difficult for me to exercise"

16. "Dermatology training has made it difficult for me to get adequate amounts of sleep"

17. "Dermatology training has made it difficult for me to engage in self-care activities"

18. "Dermatology training has made it difficult for me to maintain a romantic relationship"

19. "I feel emotionally drained from my work"

20. "I feel like my work is breaking me down"

21. "I am always / often exhausted in the morning at the thought of another day of work"

22. "My work frustrates me to a high degree"

23. "At some point during my training I have considered quitting/switching to a different career"

24. "I feel respected by my physician colleagues outside of the field of dermatology"

25. "My current location of training affords me the support structures and social connections to optimise my wellbeing"

Section 4: Imposter Phenomenon

Responses scored on a 5-point Likert scale (1 = Strongly Disagree to 5 = Strongly Agree).

26. I can give the impression that I am more competent that I am

27. I avoid evaluations if possible and have a dread of others evaluating me

28. When people praise me for something I've accomplished, I am afraid I won't be able to live up to their expectations of me in the future

29. I am afraid people important to me may find out that I'm not as capable as they think I am

30. I tend to remember the incidents in which I have not done my best more than those times I have done my best

31. At times, I feel my success has been due to some kind of luck

32. I am disappointed at times in my present accomplishments and think I should have accomplished much more

Section 5: Career Reflections and Drivers

33. Would you apply to dermatology again?

☐ Yes / ☐ No / ☐ Not sure

+2

34. Have you ever considered leaving dermatology?

☐ Yes / ☐ No / ☐ Prefer not to answer

+2

35. Which of the following would be most likely to contribute to burnout for you? (Select THREE answers at most)

Options: Transition into training , Surgical outcomes , Mini-Cex/CBD outcomes , SCE examination , Academic needs , On-call work (OOH / In-hours ), Admin load , Competition , None , Other.

+4

36. Please can you elaborate on your above answer? (Free text)

37. Which of the following would be most likely to protect you against burnout? (Select THREE answers at most)

Options: Mentorship , Educational supervision , Wellbeing e-learning , LTFT working , Mindfulness , SCE support , Balint groups , TPD access , Study Leave/Funding , Socialising , Training numbers , Admin time , Other.

+4

38. Please can you elaborate on your above answer? (Free text)

39. What is one thing you would change about your training experience? (Free text)

|  |  |  |  |  |
| --- | --- | --- | --- | --- |
|  |  |  |  |  |
|  |  |  |  |  |
|  |  |  |  |  |
|  |  |  |  |  |
|  |  |  |  |  |
|  |  |  |  |  |
|  |  |  |  |  |
|  |  |  |  |  |
|  |  |  |  |  |
|  |  |  |  |  |
|  |  |  |  |  |
|  |  |  |  |  |
|  |  |  |  |  |
|  |  |  |  |  |
|  | | | | |
|  | | | | |
